# Primary Cutaneous B-Cell Lymphoma in a Young Female

**DOI:** 10.7759/cureus.42109

**Published:** 2023-07-19

**Authors:** Zoha Aziz, Natasha Ali

**Affiliations:** 1 Medicine, Aga Khan University, Karachi, PAK; 2 Pathology & Laboratory Medicine, Aga Khan University, Karachi, PAK; 3 Oncology, Aga Khan University, Karachi, PAK

**Keywords:** b-cell lymphoma, rchop therapy, diffuse large b cell lymphoma (dlbcl), primary cutaneous b-cell lymphoma, primary cutaneous diffuse large b-cell lymphoma

## Abstract

Primary cutaneous lymphomas are a group of lymphomas that originate in the skin at the time of diagnosis. We report a case of a 45-year-old female who presented with cutaneous lesions that were unresponsive to conservative management. A biopsy was performed, which was consistent with primary cutaneous B-cell lymphoma. She received four cycles of chemotherapy and her end-of-treatment positron emission tomography (PET)-computed tomography (CT) scan showed a complete metabolic response.

## Introduction

Primary cutaneous B-cell lymphoma (CBCL) arises in the skin and shows no symptoms outside the skin at the time of diagnosis and for six months following the diagnosis [1]. According to the World Health Organization-European Organization for Research and Treatment of Cancer (EORTC) joint classification, there are three main subtypes of primary cutaneous B-cell lymphomas: primary cutaneous diffuse large B-cell lymphoma leg type, primary cutaneous follicular center lymphoma, and primary cutaneous marginal zone lymphoma [2]. CBCL is characterized by large neoplastic cells found throughout the dermis and subcutaneous tissue and mimics systemic DLBCL histologically. Hence, the standard treatment regimen of DLBCL, i.e., rituximab, cyclophosphamide, doxorubicin, vincristine, and prednisone (R-CHOP) is the treatment of choice for this type of lymphoma.

## Case presentation

**Figure 1:**
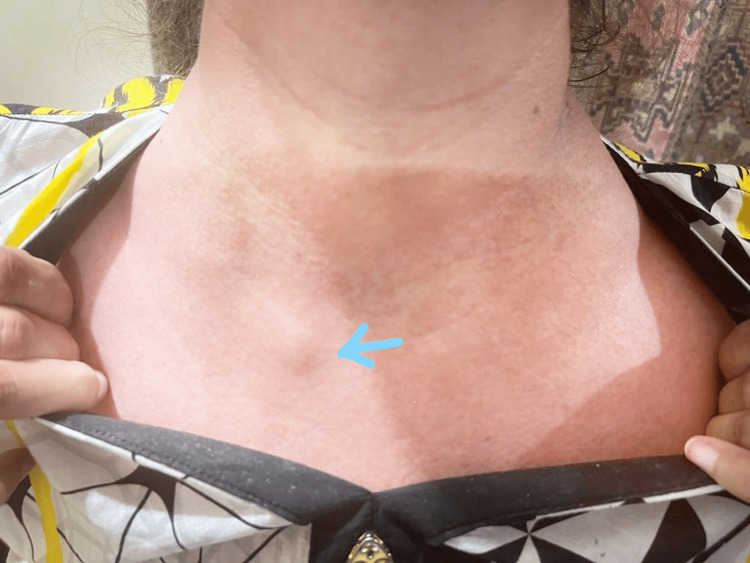
Clinical picture of cutaneous B-cell lymphoma Skin-colored, pea-sized subcutaneous nodule on the upper chest

A 45-year-old female, with a long-standing history of varicose veins, presented with a 20-day-long history of skin-colored, pea-sized swellings on her body; one above the left clavicle, one under the left ear, two under the chin, one on the upper chest (Figure 1) and one on the upper outer quadrant of the right breast in July 2022.

There was no history of fever, weight loss, or night sweats. The patient consulted a general surgeon in her hometown with concerns about pain in her right breast, swelling, and the possibility of breast cancer. She underwent a workup, which included an ultrasound of the breast and mammography. The results were, however, unremarkable. She also underwent a workup for tuberculosis, which was also negative. The surgeon diagnosed it as a case of multiple sebaceous cysts and put the patient on oral antibiotics. However, the swellings did not decrease or increase in size.

**Figure 2:**
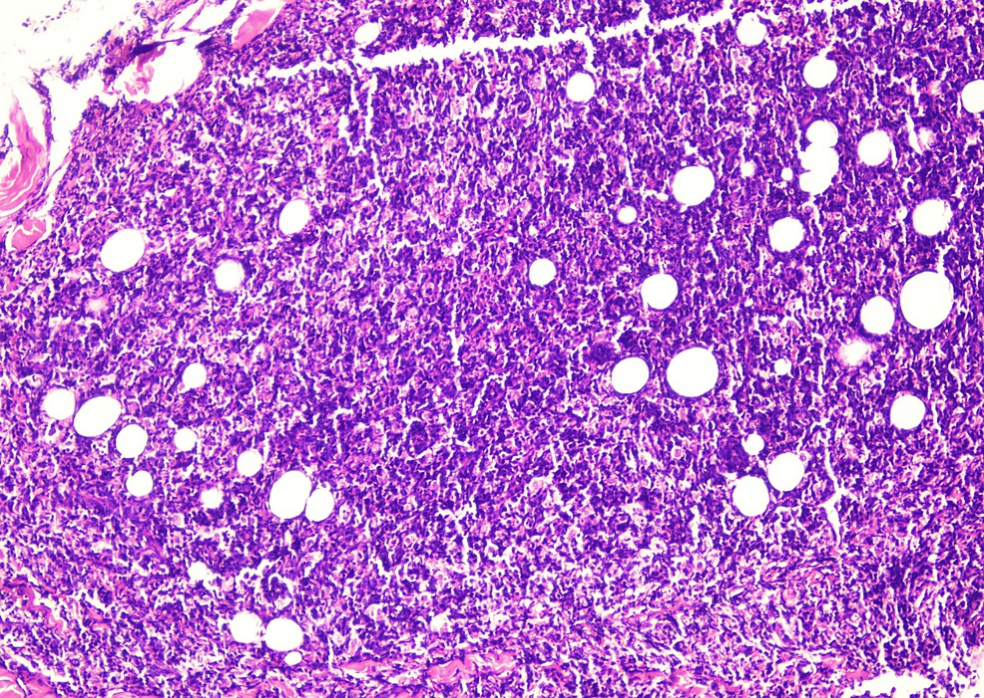
Tissue biopsy showing sheets of neoplastic cells (H & E 20x)

**Figure 3:**
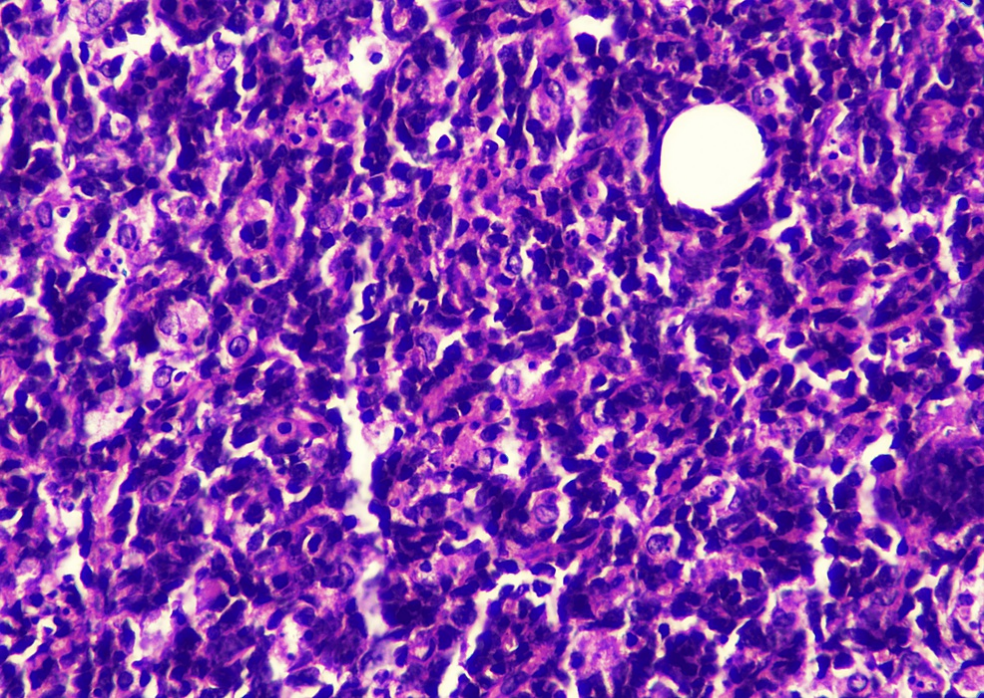
Tissue biopsy showing sheets of neoplastic cells (H & E 40x)

**Figure 4:**
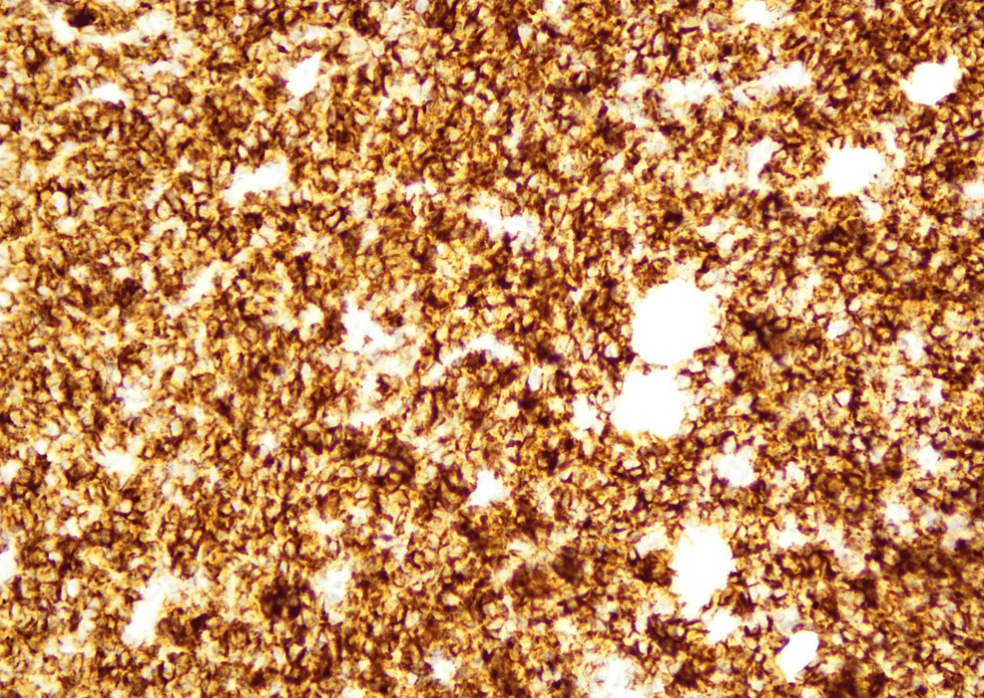
Results of immunohistochemical stains (CD20 positive 40x)

**Figure 5:**
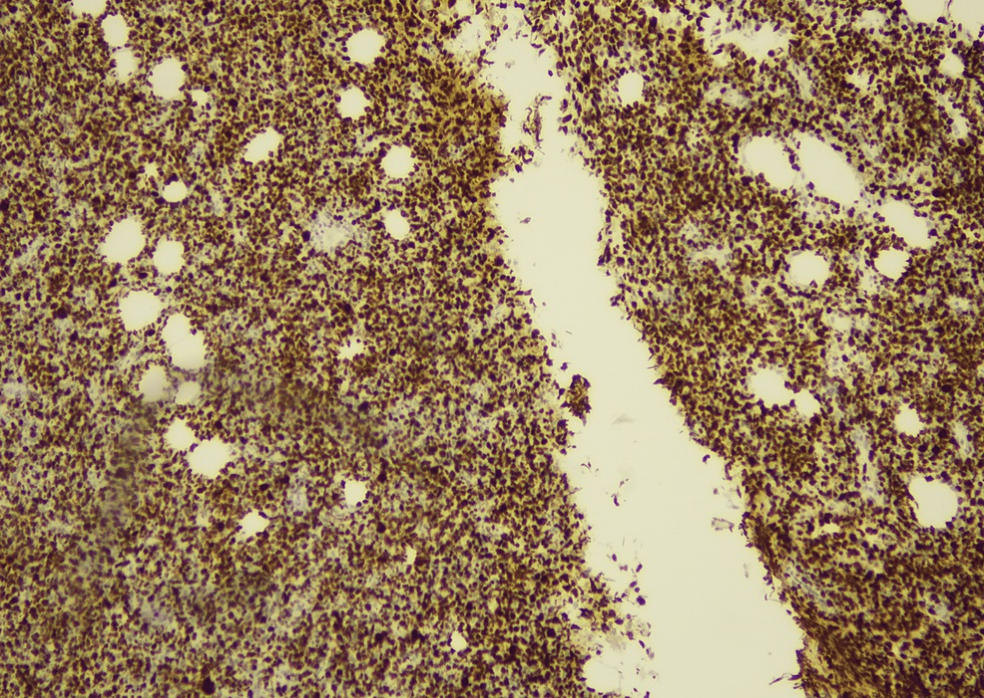
Results of immunohistochemical stains (Ki67 positive 10x)

**Figure 6:**
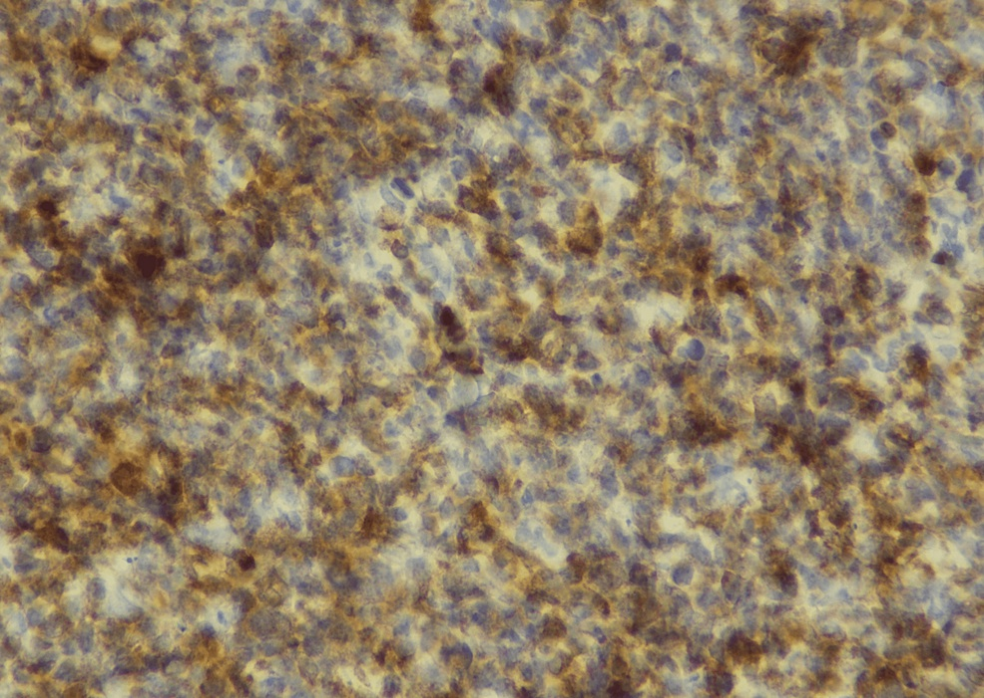
Results of immunohistochemical stains (BCL2 positive 40x)

In September 2022, an excisional biopsy of the swelling under the chin was done. Histopathological examination of the mass revealed tissue fragments lined by an unremarkable epidermis. The underlying deep fibroadipose tissue showed an infiltrate composed of sheets of neoplastic cells that were large, pleomorphic, with hyperchromatic nuclei, inconspicuous nucleoli, and moderate to scanty cytoplasm (Figures 2, 3). To deduce the presence of B-lineage differentiation, immunohistochemistry tests were conducted, which showed a reactivity pattern in neoplastic cells. CD20 (Pan B), Pax-5, Mum-1, BCL-2, KI-67, and C-MYC were found to be positive (Figures 4-6), whereas CD3 (Pan T), BCL-6, and Cyclin D-1 were found to be negative.

**Figure 7:**
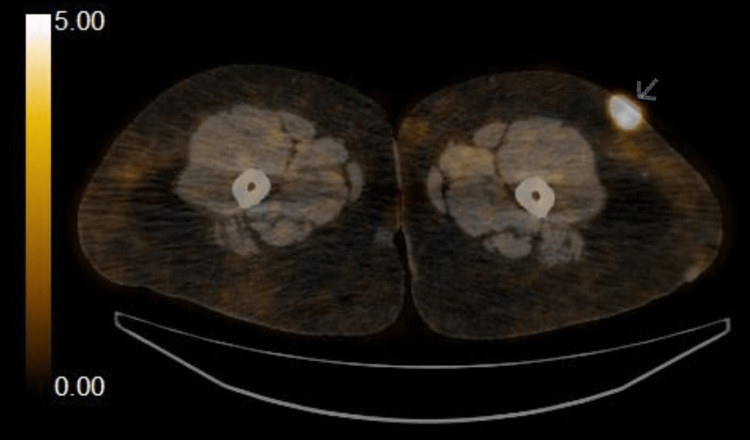
Positron emission tomography (PET) scan showing a large hypermetabolic nodule on the left thigh anterolaterally (28 x 17 mm)

The patient was immediately referred to a tertiary care hospital for further workup and management, where a diagnosis of primary cutaneous large B-cell lymphoma, leg type was made. A PET scan was performed, which showed evidence of numerous hypermetabolic subcutaneous nodules all over the body, the largest over the left thigh anterolaterally (Figure 7).

There was no evidence of hypermetabolic nodal, hepatic, adrenal, pulmonary, or bony deposits on the scan.

The patient was treated with the R-CHOP (rituximab, cyclophosphamide, vincristine, doxorubicin, and prednisone) regimen, and she received a total of four cycles. Interim PET scan, after six months of chemotherapy, revealed interval normalization of hypermetabolic subcutaneous nodules while the end-of-treatment scan was consistent with complete metabolic response. She is due for a follow-up in three months.

## Discussion

Primary cutaneous B-cell lymphoma (CBCL) is a lymphoma that originates in the skin and exhibits no symptoms external to the skin at the time of diagnosis and for six months subsequently [1]. According to the World Health Organization-European Organization for Research and Treatment of Cancer (EORTC) joint classification, there are three main subtypes of primary cutaneous B-cell lymphomas: primary cutaneous follicular center lymphoma, primary cutaneous diffuse large B-cell lymphoma leg type, and primary cutaneous marginal zone lymphoma. The updated 2017 WHO classification includes EBV-positive mucocutaneous ulcer (EBV-MCU) as a new provisional entity among uncommon B-cell proliferations. Primary cutaneous follicular lymphoma is the most common subtype in this classification [2,3].

The presence of B-lineage differentiation can be verified by using methods like immunohistochemistry (IHC) or flow cytometry. The former requires the comparison of a T-cell marker and at least one B-cell marker such as CD3 and CD20. A second B-cell marker like CD19 and CD21 should be examined if CD20 is negative because 1% to 2% of B-cell lymphomas do not express CD20. For instance, CD20 is typically negative in plasmablastic lymphoma and ALK-positive large B-cell lymphomas [4]. In our report, we are primarily focusing on CBCL. Lower limb tumors that are rapidly spreading can lead to a diagnosis of CBCL, leg type, as seen in our patient. In some situations, lymphoma may spread outside of the skin and damage other parts of the skin as well. However, in our patient, the lymphoma was extranodal with no systemic involvement. CBCL is characterized by large cells called centroblasts and immunoblasts, which are found throughout the dermis and subcutaneous tissue. CBCLs have high quantities of BCL-2 due to gene amplification. The occurrence of an isolated c-MYC translocation is rare. Albeit, dual expression of BCL-2 and c-MYC occurs in two-thirds of cases and is linked to worse overall survival rates. CBCL, leg-type, cells have a gene expression pattern resembling that of activated B cells and are frequently positive for MUM-1/IRF-4 and BCL-6 while being negative for CD10 [4]. MYD88 mutations, which are commonly found in activated B-cell diffuse large B-cell lymphoma (ABC-DLBCL), are also present in this type of lymphoma, indicating a shared involvement of NF-κB activation. The B-cell receptor's aberrant signaling is a causative factor of the disorder, as Staphylococcal superantigen binding sites within the immunoglobulin heavy-chain variable (IGHV) are unaffected despite the use of somatically hypermutated IGHV regions. Generally, CBCL, leg type, is more comparable to systemic DLBCL [1,4]. In comparison to PCFCL, CBCL, leg type, has a worse prognosis and tends to migrate to other body regions. Differential immunophenotypes and genetic aberrations can be used to distinguish between the two types of lymphomas in addition to clinical and histological criteria [5].

It is interesting to note that, unlike previously documented instances of primary cutaneous B-cell Lymphoma, this case can be inferred as the first occurrence of a patient presenting primarily with neck swellings devoid of any accompanying redness or itching. Furthermore, no previous medical history or examination revealed any other cause of such swellings such as HIV or tuberculosis. As a result, if prompt action is not taken, such a condition may be ignored by the patient or misdiagnosed by the physician.

A key aspect in the treatment approach of all prior known CBCLs was recognizing that the natural course of CBCL is quite similar to that of systemic DLBCL. As a result, the standard treatment regimen of rituximab, cyclophosphamide, doxorubicin, vincristine, and prednisone (R-CHOP), with or without radiation therapy, is always the primary treatment strategy [6]. Our patient responded very well to this treatment and achieved a complete metabolic response after completion of therapy as documented on PET scan results.

## Conclusions

Primary cutaneous diffuse large B-cell lymphoma, leg type, is a rare type of primary cutaneous lymphoma. It originates primarily in the skin and mimics systemic diffuse large B-cell lymphoma. This is a case report of a rare B-cell lymphoma in a young female who responded well to standard chemotherapy and achieved complete metabolic response at the end of therapy and has remained symptoms free ever since.

## References

[1] Demierre MF, Kerl H, Willemze R (2003). Primary cutaneous B-cell lymphomas: a practical approach. Hematol Oncol Clin North Am.

[2] Willemze R, Cerroni L, Kempf W, Berti E, Facchetti F, Swerdlow SH, Jaffe ES (2019). The 2018 update of the WHO-EORTC classification for primary cutaneous lymphomas. Blood.

[3] Pandolfino TL, Siegel RS, Kuzel TM, Rosen ST, Guitart J (2000). Primary cutaneous B-cell lymphoma: review and current concepts. J Clin Oncol.

[4] King JF, Lam JT (2020). A practical approach to diagnosis of B-cell lymphomas with diffuse large cell morphology. Arch Pathol Lab Med.

[5] Kempf W, Zimmermann AK, Mitteldorf C (2019). Cutaneous lymphomas—an update 2019. Hematol Oncol.

[6] Wilcox RA (2018). Cutaneous B-cell lymphomas: 2019 update on diagnosis, risk stratification, and management. Am J Hematol.

